# Linagliptin ameliorated interleukin-29-induced reduction of extracellular matrix genes through the nuclear factor erythroid 2-related factor 2 (Nrf2)/sry-type high-mobility-group box (SOX)-9 axis in an *in vitro* study on C-28/I2 chondrocytes

**DOI:** 10.1080/21655979.2022.2031407

**Published:** 2022-01-26

**Authors:** Ying Li, Peng Zhan, Qiang Wang, Minghua Zhang, Shiming Huang, Dongfeng Chen

**Affiliations:** Department of Joint Surgery and Sports Medicine, Longyan First Affiliated Hospital of Fujian Medical University, Longyan, China

**Keywords:** Osteoarthritis, C-28/I2 chondrocytes, IL-29, Nrf2, SOX-9, DPP-4 inhibitors

## Abstract

Osteoarthritis (OA) is a severe orthopedic disease commonly observed in the elderly population and is closely related to the degradation of extracellular matrix (ECM) in cartilage tissues. Interleukin-29 (IL-29) is a cytokine that has been recently linked with the progression of OA. However, the physiological roles of IL-29 in ECM genes and function are unknown. Linagliptin is a novel dipeptidyl peptidase-4 (DPP-4) inhibitor recently reported to exert significant anti-inflammatory properties. In this study, we used IL-29 to stimulate C-28/I2 chondrocytes to build an inflammatory injury model. We aimed to investigate the protective effect of Linagliptin on IL-29-induced degradation of ECM. We found that IL-29 stimulation reduced the expressions of *Col2a1* and *Acan* in C-28/I2 chondrocytes, and this effect was mediated by SRY-related high-mobility group box gene-9 (SOX-9), as we showed that overexpression of SOX-9 could rescue the reduction of *Col2a1* and *Acan*. Interestingly, we found that IL-29 stimulation pronouncedly promoted the expression of DPP-4. Treatment with 100 nM of the DPP-4 inhibitor Linagliptin ameliorated IL-29-induced expressions of SOX-9, *Col2a1*, and *Acan*. Lastly, the nuclear level of nuclear factor erythroid 2-related factor 2 (Nrf2) was dramatically declined in IL-29-challenged chondrocytes and the protective effects of Linagliptin on the expressions of SOX-9, *Col2a1*, and *Acan* were abolished by the knockdown of Nrf2. Taken together, our data reveal that Linagliptin ameliorated IL-29-induced reduction of ECM genes partially through the Nrf2/SOX-9 axis in C-28/I2 chondrocytes. Further *in vivo* and clinical studies will be done to clarify the protective benefits of Linagliptin in OA.

## Graphical abstract

**Figure:**
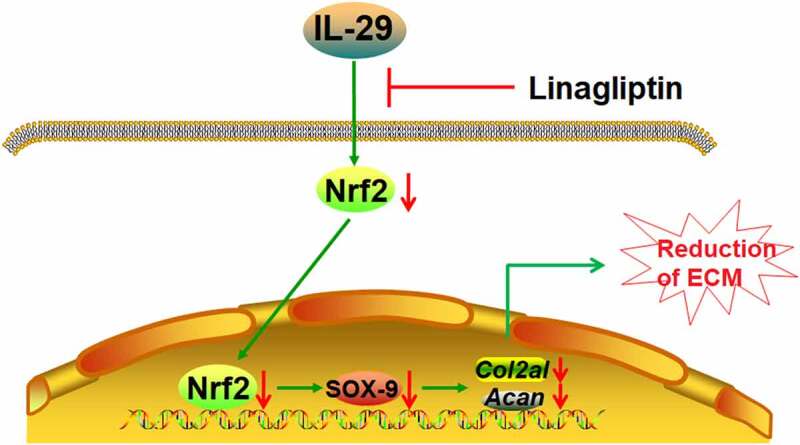

## Introduction

Osteoarthritis (OA) is a degenerative disease in the field of orthopedics with high morbidity. It impacts approximately 10% of the elderly population over the age of 60. Great pain in the knee or hip joint induced by OA is generally accompanied by dysfunction and significantly impacts the life quality of elderly patients. However, rare effective therapies for the treatment of OA are available in the clinic. Currently, symptomatic treatment, such as non-steroid anti-inflammatory drugs (NSAIDs) usage, is applied for OA patients, whereas medications to reverse cartilage damage and progression of OA are scarce [1–4]. The most significant characteristic of OA is the progressive disruption of cartilage tissues and the essence of its treatment is to maintain the normal metabolism of chondrocytes and prevent the disruption of cartilage homeostasis [5–7]. As the main cell type in cartilage tissues, chondrocytes are responsible for the synthesis of extracellular matrix (ECM) components such as collagens and aggrecan, which provide the mechanical support for cartilage tissues for articular movements [8,9]. *Col2a1* and *Acan* encode the expression of ECM components and are widely reported to be downregulated in OA cartilages [10,11]. They can, however, be positively regulated by the transcriptional factor, SOX-9 [12]. Therefore, it is of great significance to protect ECM components from being degraded when treating OA. Nuclear factor erythroid 2-related factor 2 (Nrf2) has been demonstrated to play a critical role in the development of OA. Kong *et al*. found that the activity of Nrf2 could inhibit IL-1β–induced overexpression of MMP-1, −3, and −13 [13]. Furthermore, Nrf2 was found to suppress PGE_2_ and NO production by regulating the NF-κB pathway [14]. In 2013, a study showed that Sulforaphane, a potent activator of Nrf2, decreased cartilage degradation in a murine post-traumatic model of OA [15]. This finding indicated the potential protective benefits of Nrf2 on the progression of OA.

The synthesis of cartilage ECM can be suppressed by the excessive production of pro-inflammatory factors, such as interleukin-1β (IL-1β) and tumor necrosis factor-α (TNF-α). Recently, a novel pro-inflammatory factor, IL-29, had been found to be responsible for the enhanced synovial inflammation and cartilage disruption in OA progression [16]. However, the biological functions of IL-29 in the synthesis of cartilage ECM have not been reported before. In the present study, IL-29 will be used to induce the *in vitro* injury model on chondrocytes to investigate the underlying mechanism.

Linagliptin, a novel inhibitor of DPP-4, is applied for the treatment of type II diabetes with the advantage of high affinity to cell membranes in serum and tissues. Therefore, its half-life is relatively long (>100 hours) [17]. Linagliptin suppresses the degradation of the glucose-dependent insulinotropic polypeptide (GIP) and glucagon-like peptide (GLP-1) by reversibly binding with DPP-4, which further contributes to the decreased level of blood glucose [18]. International clinical trials have revealed that Linagliptin showed significant efficacy against type II diabetes with high safety and toleration [19]. Recently, the significant anti-inflammatory effect of Linagliptin has been widely reported. In 2014, Nakamura YY *et al*. showed that Linagliptin inhibited the expressions of IL-6 and prostaglandin E2 (PGE_2_), which play a pivotal role in inflammatory responses, in hemodialysis patients with type 2 diabetes [20]. Furthermore, Jo CH *et al*. showed new evidence that Linagliptin suppressed NLRP3 inflammasome activation in an *in vivo* rat doxorubicin nephropathy model [21]. Importantly, in an *in vivo* rat carotid balloon injury model, Linagliptin activated the Nrf2 antioxidant pathway to counter the intimal hyperplasia caused by vascular injury. These findings indicate the anti-inflammatory benefits of Linagliptin, are not only on the diabetes-related disease but also on other diseases. In the present study, the protective effect of Linagliptin on IL-29-induced degradation of ECM components will be investigated to provide preliminary evidence for treating OA.

## Materials and methods

### Cell culture

Human C-28/I2 chondrocytes were obtained from Shanghai Y-S Biotechnology (Shanghai, China) and cultured in the DMEM/F12 medium supplemented with 10% FBS under 5% CO_2_ and 37°C conditions. Cells were stimulated with 0, 10, 15 ng/mL IL-29 for 24 hours to induce inflammatory injury. Cells were stimulated with 15 ng/mL IL-29 [22] in the presence or absence of Linagliptin (100 nM) for 24 hours to investigate the protective effect of Linagliptin.

### Transfection

The special siRNA against Nrf2 and Lentiviral SOX-9 was purchased from Santa Cruz Biotechnology, USA. 1 × 10^5^ cells were seeded onto 6-well cell culture plates. After reaching 60–80% of confluence, 20 μM nonspecific siRNA and Nrf2 siRNA were transfected to C-28/I2 chondrocytes using 3 μL Lipofectamine RNAiMAX reagent (Thermo Fisher Scientific, USA). Lentiviral SOX-9 was transduced to C-28/I2 chondrocytes in accordance with the manufacturer’s instructions. The overexpression and knockdown efficacy were verified using Western blotting assay [23].

### Real-time PCR analysis

The TRIZOL reagent (Invitrogen, California, USA) was utilized for the extraction of total RNAs from the chondrocytes in each group. 2 μg RNA was then further reverse‐transcribed to cDNA using the TaqMan RNA reverse transcription kit (Invitrogen, California, USA). The RT-PCR was conducted with SYBR Green Real‐time PCR Master Mix (Roche Diagnostics, Basel, Switzerland), the conditions of which were 94°C for 5 min, 30 cycles of 94°C for 30 sec, and 58–61°C for 30 sec, followed by 72°C for 2 min. GAPDH was used for the normalization of relative expression of target genes, which was calculated using the 2^−ΔΔCt^ method [24]. The following primers were used in this study:

*Col2a1*, forward 5’-TGGACGATCAGGCGAAACC-3’, reverse 5’-GCTGCGGATGC TCTCAATCT-3’; *Acan*, forward 5’- ACTCTGGGTTTTCGTGACTCT-3’, reverse 5’- ACACTCAGCGAGTTGTCATGG-3’; SOX-9, forward 5’-AGGAGAACCCCAAG ATGCAC-3’, reverse 5’-GAGGCGTTTTGCTTCGTCAA-3’; DPP-4, forward 5’-GGG TCACATGGTCACCAGTG-3’, reverse 5’-TCTGTGTCGTTAAATTGGGCATA-3’; GAPDH forward 5’-GAGAAGGCTGGGGCTCATTT-3’, reverse 5’-AGTGATGGC ATGGACTGTGG-3’.

### Western blotting assay

The cell lysis buffer (Cell Signaling Technology, California, USA) containing protease and phosphatase inhibitor cocktail (Abcam, USA) was used for the extraction of total proteins from the chondrocytes, followed by quantification with a BCA kit (Shanghai Ze Ye Biotechnology Co., Ltd, Shanghai, China). After loading approximately 30 μg protein, 12% SDS PAGE was used to separate the proteins, which were further transferred to the PVDF membrane (Cell Signaling Technology, California, USA). Then, the protein-loaded PVDF membrane was mixed with 5% skim milk to block the nonspecific binding proteins, followed by incubation in the solution of primary antibody against SOX-9 (1:1000, R&D, Minnesota, USA), Nrf2 (1:2000, R&D, Minnesota, USA), DPP-4 (1:2500, R&D, Minnesota, USA), Lamin B1 (1:500, R&D, Minnesota, USA) and GAPDH (1:800, R&D, Minnesota, USA). The membrane was subsequently incubated with the secondary antibody (R&D, Minnesota, USA). Lastly, the bands were visualized using the enhanced chemiluminescence (ECL) kits (Cell Signaling Technology, California, USA) on a Tanon-2500 imaging system (Shanghai, China), followed by quantifying the relative expression level of target proteins with Image J software ^25^.

### Immunostaining assay

Chondrocytes were collected and washed using the PBS buffer and subsequently fixed with 4% paraformaldehyde on a microslide for 10 min, followed by 3 washes and treated with triton-100 for 10 min. After blocking using the 5% goat serum, cells were incubated with the primary antibody against SOX-9 (R&D, Minnesota, USA) at 37°C for 1.5 h, followed by incubation with a solution diluted with secondary antibody (R&D, Minnesota, USA) at 37°C for 45 min. Lastly, the slides were sealed using nail polish after adding the DAPI solution. The fluorescence was observed under the fluorescence microscope (KEYENCE, Tokyo, Japan).

### Statistical analysis

Data were analyzed using the GraphPad Prism 6.0 and were presented as mean ± standard error of measurement (S.E.M). The T-test was used to compare two independent data and the data among groups were compared using the analysis of variance (ANOVA) method followed by Tukey’s post-hoc test, while *p < 0.05* was taken as a significant difference.

## Results

Firstly, we found that exposure to IL-29 reduced the expressions of Col2a1 and Acan genes, mediated by inhibition of SOX-9 in a dose-dependent manner. IL-29 increased the expression of DPP-4. Interestingly, treatment with the DPP-4 inhibitor Linagliptin restored the effects of IL-29. Additionally, we found that silencing of Nrf2 abolished the protective benefits of Linagliptin, indicating that the beneficial function of Linagliptin against IL-29-induced reduction of ECM was mediated by Nrf2.

### *IL-29 reduced* Col2a1 *and* Acan *genes, as well as the expression of SOX-9 in human C-28/I2 chondrocytes*

**Figure 1.**
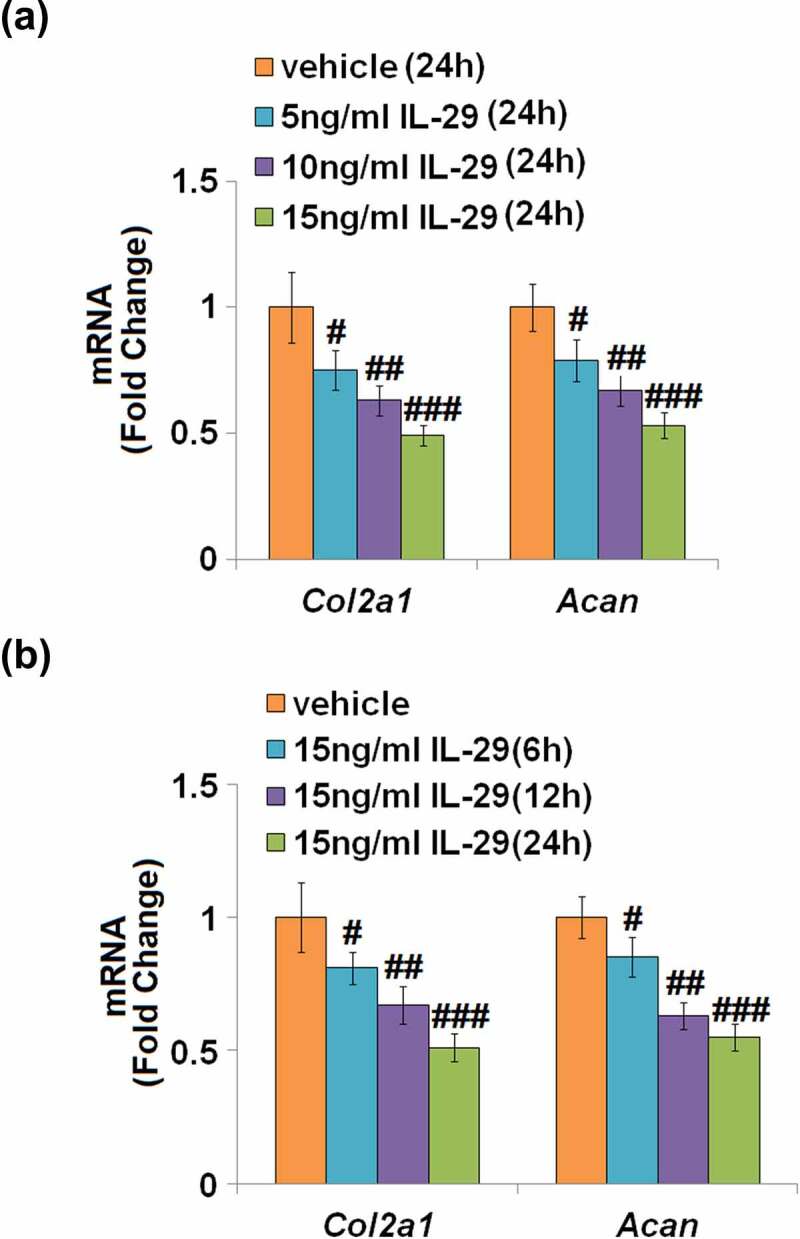
Interleukin-29 reduced *Col2a1* and *Acan* genes in human C-28/I2 chondrocytes. (a). Cells were stimulated with 0, 5, 10, 15 ng/mL for 24 hours. *Col2a1* and *Acan* mRNA were measured; (b). Cells were stimulated with 15 ng/mL IL-29 for 6, 12, and 24 hours. *Col2a1* and *Acan* mRNA were measured (#, ##, ###, *P* < 0.05, 0.01, 0.005 vs. vehicle).

**Figure 2.**
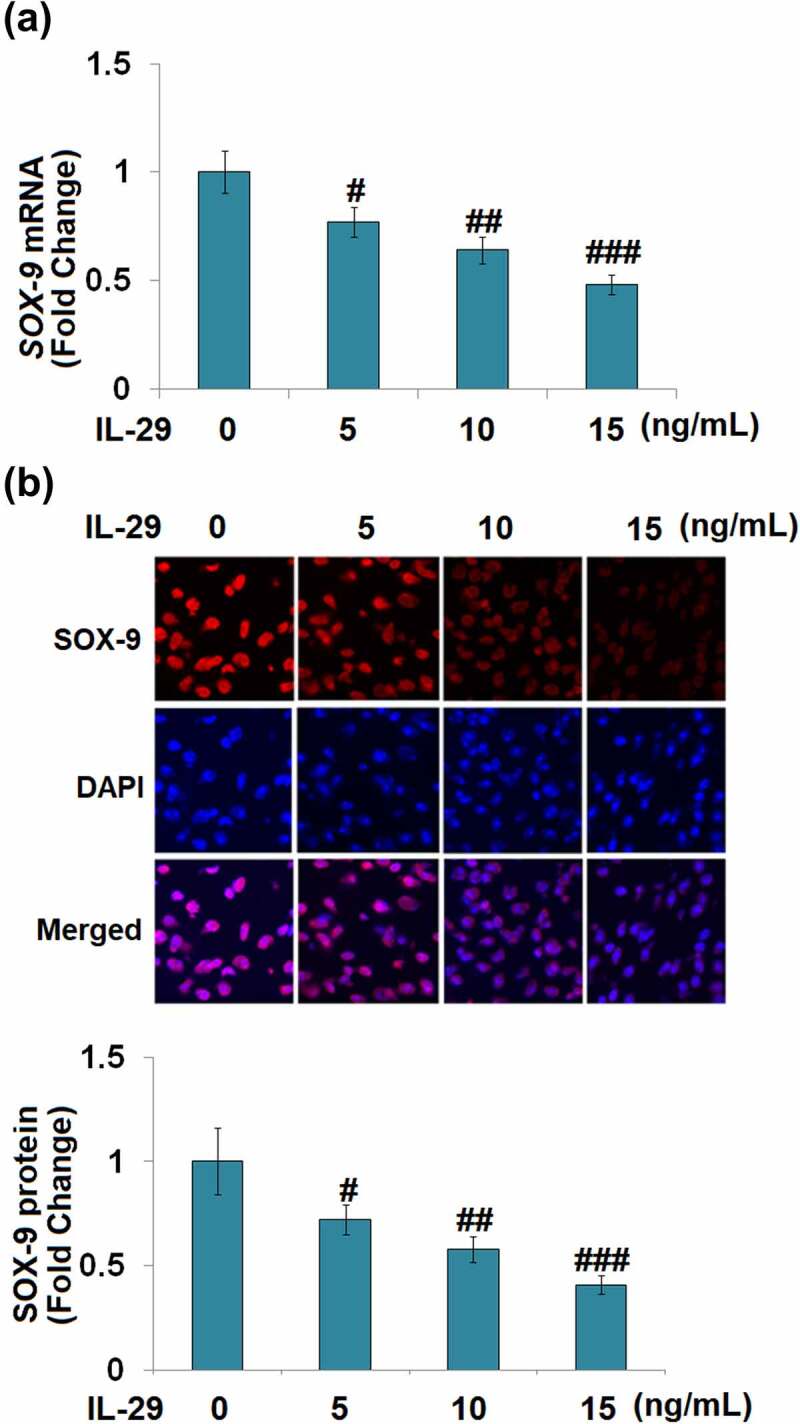
Interleukin-29 reduced the SRY-related high-mobility group box gene-9 expression in human C-28/I2 chondrocytes. Cells were stimulated with 0, 5, 10, 15 ng/mL IL-29 for 24 hours. (a). *SOX-9* mRNA; (b). Immunostaining of SOX-9 protein (#, ##, ###, *P* < 0.05, 0.01, 0.005 vs. vehicle).

As shown in Figure 1a, the expression levels of *Col2a1* and *Acan* mRNA were reduced by IL-29 in a dose-dependent manner. As shown in Figure 1b, *Col2a1* and *Acan* were downregulated by IL-29 in a time-dependent manner. Interestingly, stimulation with IL-29 also dose-dependently reduced the expression of SOX-9, an important transcriptional factor governing the expressions of *Col2a1* and *Acan*, both at the mRNA (Figure 2a) and protein levels (Figure 2b).

### *Overexpression of SOX-9 restored IL-29-induced reduction of* Col2a1 and Acan *genes*

**Figure 3.**
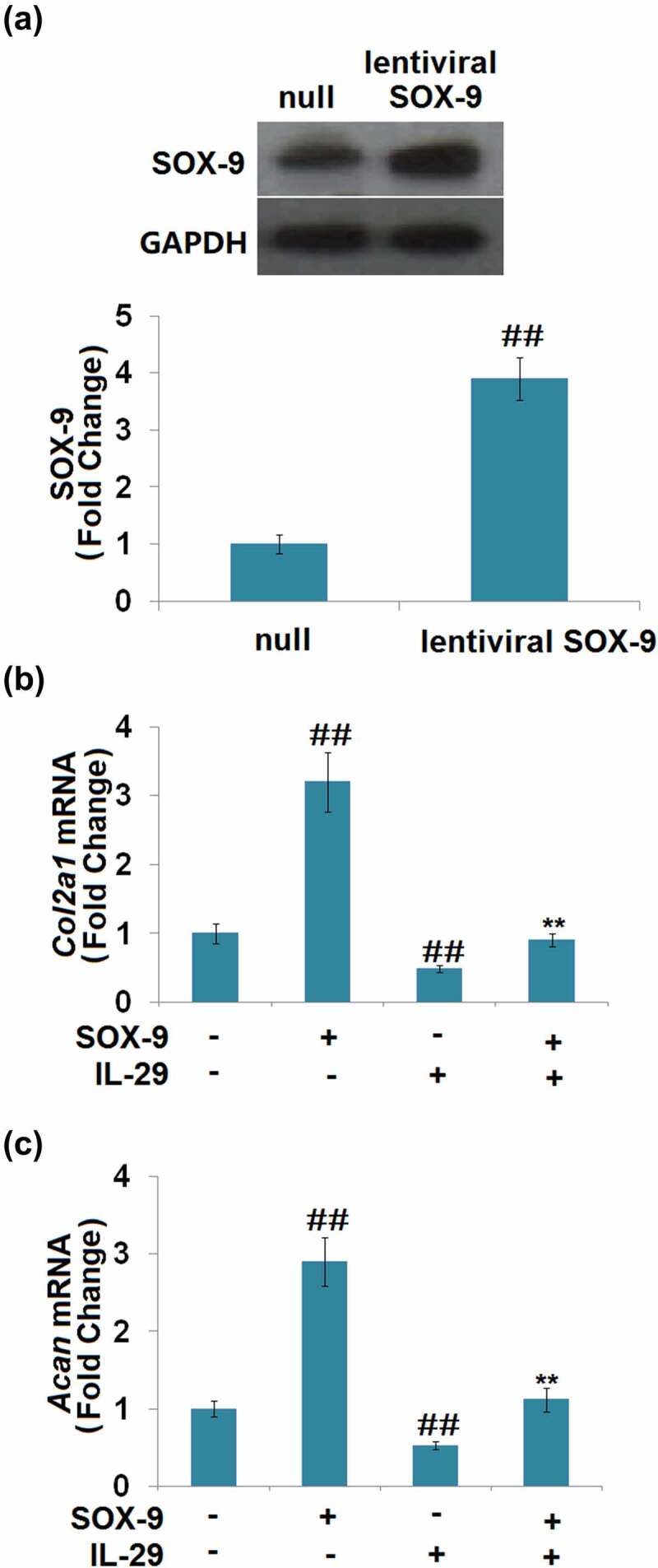
Overexpression of SRY-related high-mobility group box gene-9 restored interleukin-29- induced reduction in *Col2a1* and *Acan* gene. Cells were transduced with lentiviral SOX-9, followed by stimulation with 15 ng/mL IL-29 for 24 hours. (a). Western blot analysis revealed successful overexpression of SOX-9; (b). *Col2a1* mRNA; (c). *Acan* mRNA (##, *P* < 0.01 vs. vehicle; **, *P* < 0.01 vs. IL-29).

As shown in Figure 3a, SOX-9 was found to be significantly upregulated in lentiviral SOX-9 transduced chondrocytes, which proved the successful overexpression of SOX-9 in the chondrocytes. Compared to the vehicle group, the expression levels of *Col2a1* and *Acan* (Figure 3b–c) were significantly elevated by the overexpression of SOX-9 and suppressed by IL-29. In addition, the declined expression levels of *Col2a1* and *Acan* in IL-29-stimulated chondrocytes were dramatically reversed by the overexpression of SOX-9. These findings suggest that the inhibitory effects of IL-29 in *Col2a1 and Acan* genes expression are mediated by SOX-9.

### IL-29 increased the expression of DPP-4 in human C-28/I2 chondrocytes

**Figure 4.**
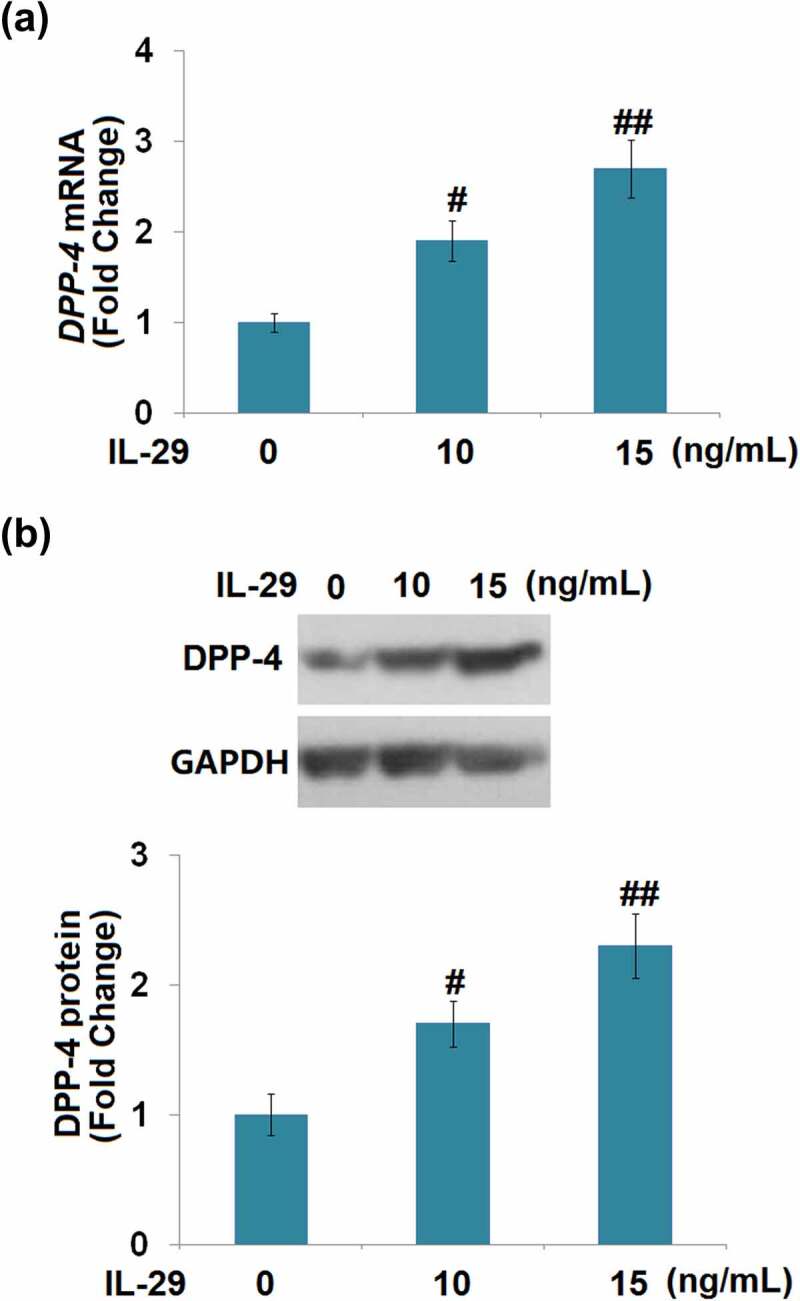
Interleukin-29 increased the expression of dipeptidyl peptidase-4 in human C-28/I2 chondrocytes. Cells were stimulated with IL-29 (0, 10, 15 ng/mL) for 24 hours. (a). DPP-4 mRNA expression; (b). DPP-4 protein expression (#, ##, *P* < 0.05, 0.01 vs. vehicle).

The effects of IL-29 on DPP-4 expression have not been reported before. We found that the expression level of DPP-4 was significantly elevated by the stimulation with IL-29 in a dose-dependent manner (Figure 4a–b). These findings implicate that DPP-4 might be relevant to the biological function of IL-29 in chondrocytes.

### *Linagliptin ameliorated IL-29-induced decrease in SOX-9*, Col2a1 *and* Acan *genes*

**Figure 5.**
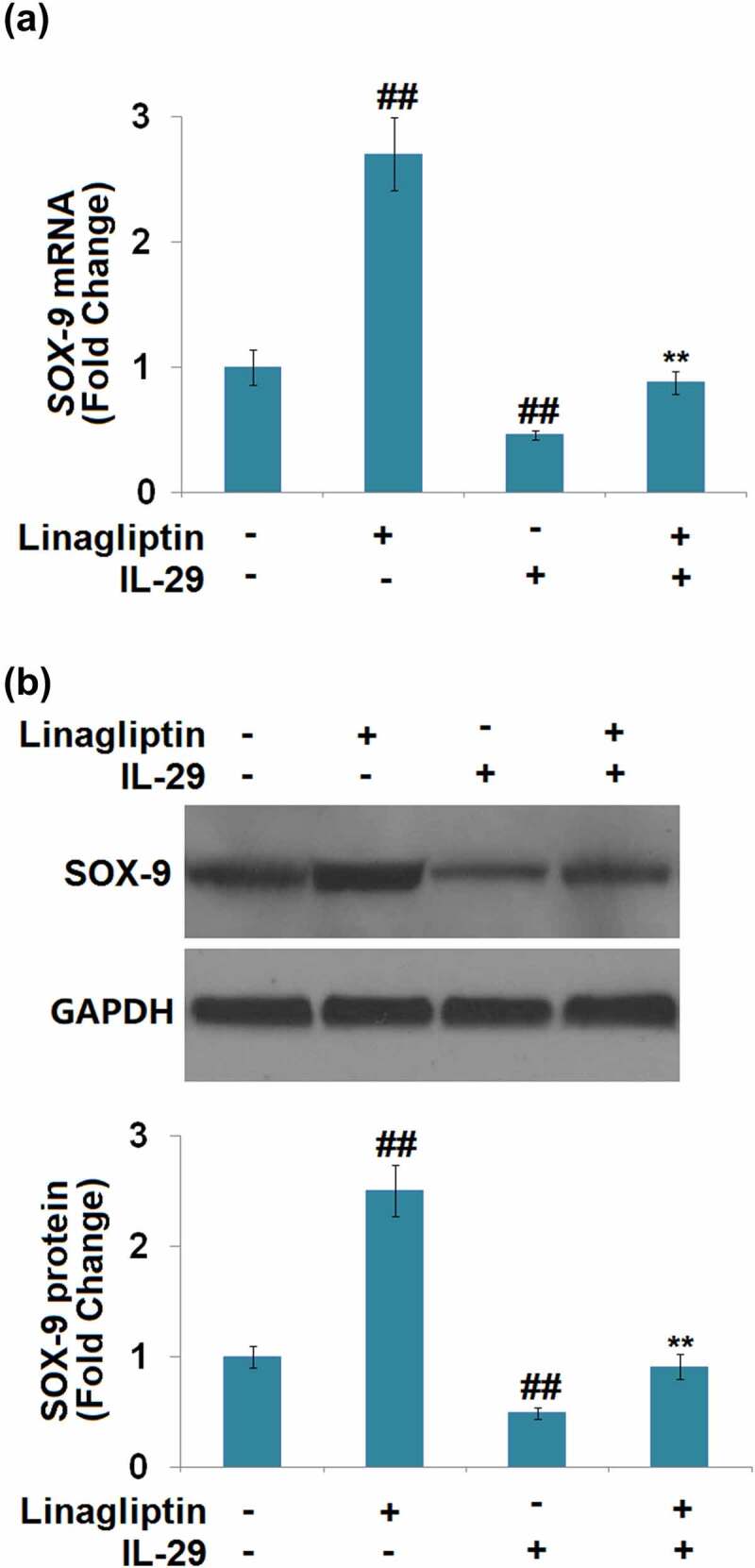
Linagliptin ameliorated interleukin-29-induced decrease in SRY-related high-mobility group box gene-9. Cells were incubated with 15 ng/mL IL-29 in the presence or absence of Linagliptin (100 nM) for 24 hours. (a). Molecular structure of Linagliptin; (b). SOX-9 mRNA; (c). SOX-9 protein (##, *P* < 0.01 vs. vehicle; **, *P* < 0.01 vs. IL-29).

We found that compared to the vehicle group, SOX-9 (Figure 5a–b) was greatly upregulated by Linagliptin and downregulated by IL-29. The declined expression level of SOX-9 in IL-29-treated chondrocytes was significantly elevated by the introduction of Linagliptin.

**Figure 6.**
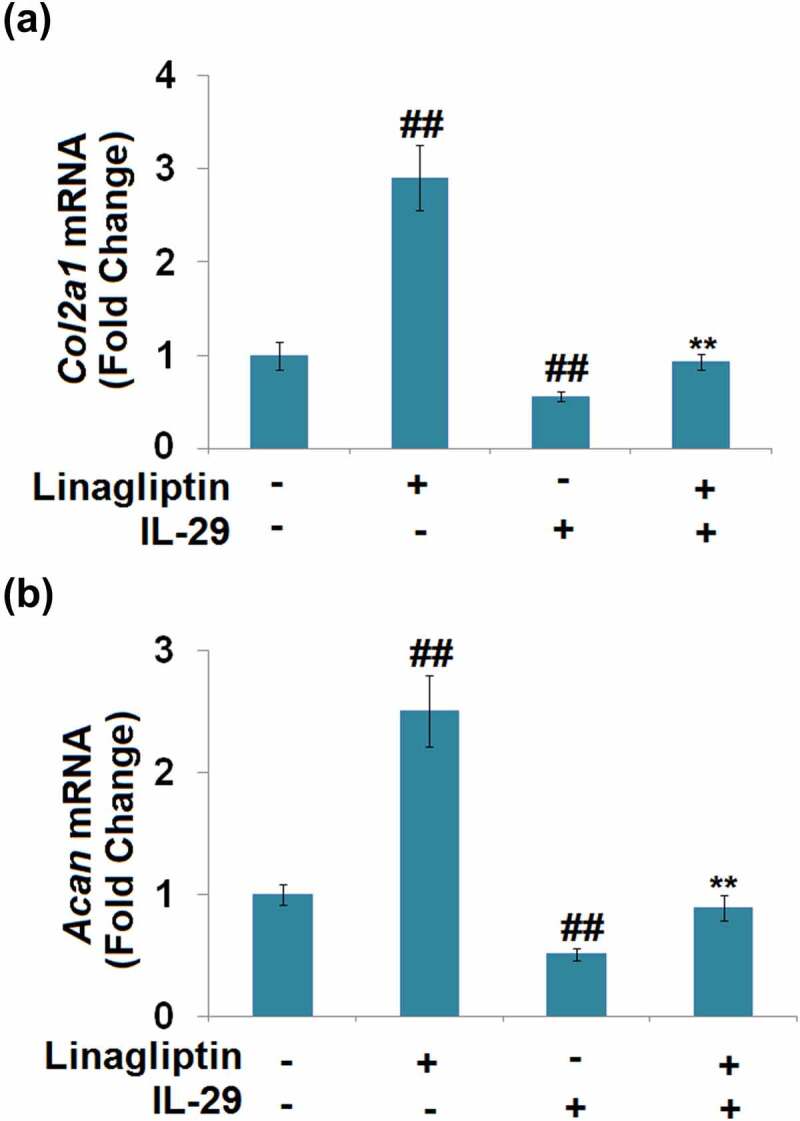
Linagliptin attenuated interleukin-29-induced decrease in *Col2a1* and *Acan* genes. Cells were incubated with 15 ng/mL IL-29 and Linagliptin (100 nM) for 24 hours. (a). *Col2a1* mRNA; (b). *Acan* mRNA (##, *P* < 0.01 vs. vehicle; **, *P* < 0.01 vs. IL-29).

We further measured the profiles of the synthesis of ECM components. As shown in Figure 6a–b, compared to the vehicle group, the expression levels of *Col2a1* and *Acan* were dramatically promoted by the treatment with Linagliptin and decreased by the stimulation with IL-29.

### *The protective effects of Linagliptin in the expressions of SOX-9*, Col2a1, *and* Acan *are dependent on Nrf2*

**Figure 7.**
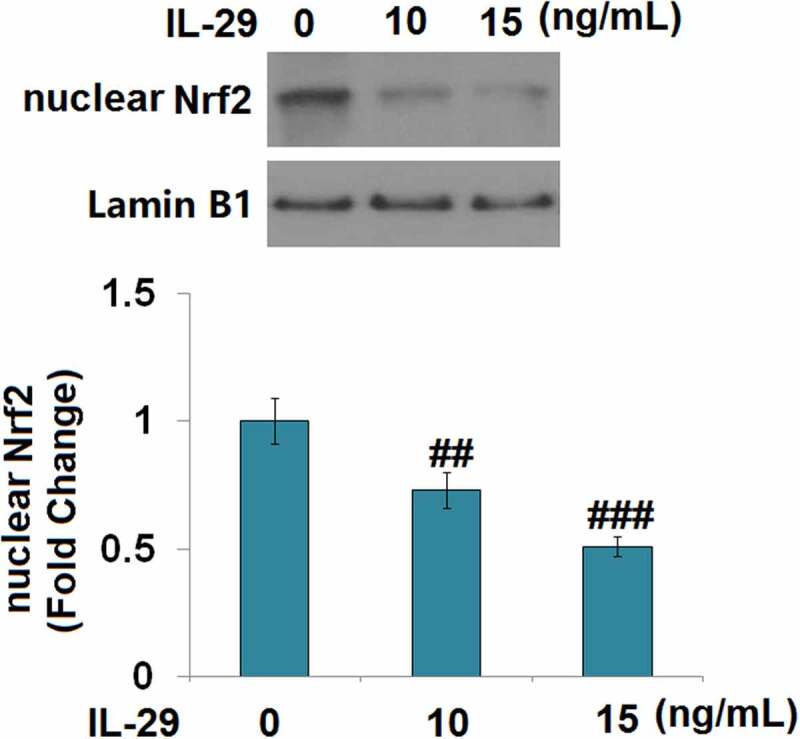
Interleukin-29 inactivated nuclear factor erythroid 2-related factor 2 signaling. Cells were stimulated with 0, 10, 15 ng/mL IL-29 for 6 hours. Protein levels of nuclear Nrf2 were measured (##, ###, *P* < 0.01, 0.005 vs. vehicle).

**Figure 8.**
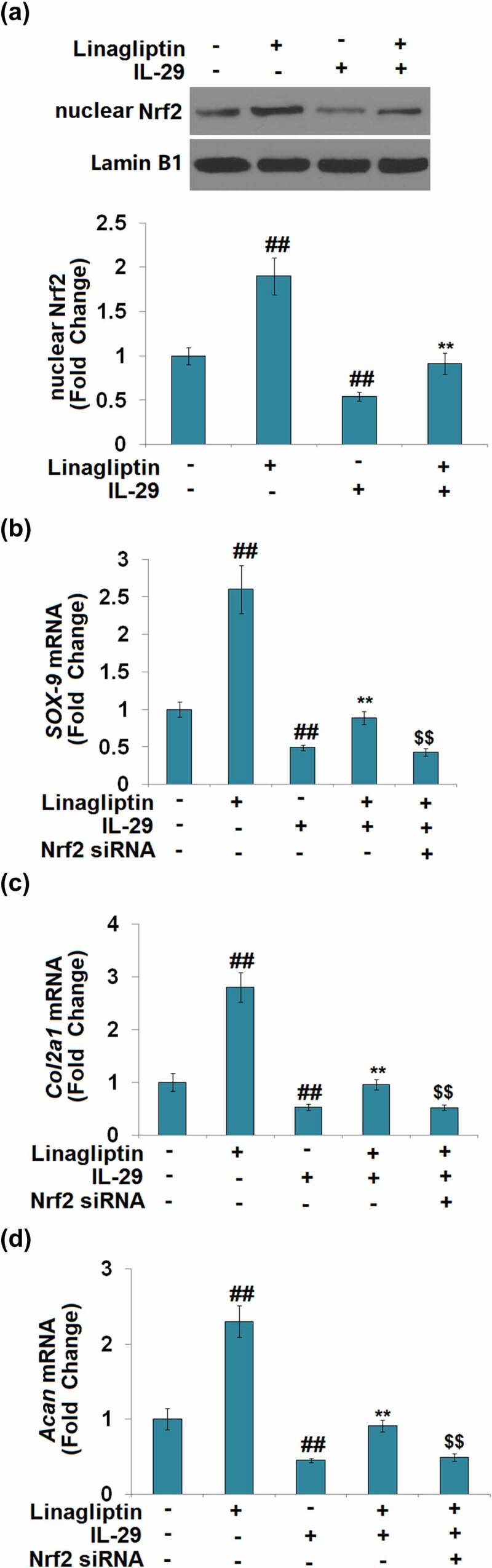
The protective effects of Linagliptin in the expression of SRY-related high-mobility group box gene-9, *Col2a1*, and *Acan* were dependent on nuclear factor erythroid 2-related factor 2. (a). Cells were stimulated with 15 ng/mL IL-29 in the presence or absence of Linagliptin (100 nM) for 24 hours. Levels of nuclear Nrf2 were measured. (b–d). Cells were transfected with Nrf2 siRNA, followed by stimulation with 15 ng/mL IL-29 and Linagliptin (100 nM) for 24 hours. SOX-9 mRNA, *Col2a1* mRNA, and *Acan* mRNA were measured (##, *P* < 0.01 vs. vehicle; **, *P* < 0.01 vs. IL-29; $$, *P* < 0.01 vs. IL-29+ Linagliptin).

As shown in Figure 7, we found that the concentration of IL-29 was inversely proportional to the expression level of nuclear Nrf2. Compared to the vehicle group, the nuclear level of Nrf2 (Figure 8a) was significantly elevated by Linagliptin and decreased by the stimulation with IL-29. In addition, the declined level of nuclear Nrf2 in IL-29-treated chondrocytes was greatly reversed by Linagliptin. Then, cells were transfected with Nrf2 siRNA, followed by stimulation with 15 ng/mL IL-29 in the presence or absence of Linagliptin (100 nM) for 24 hours. As shown in Figure 8b–d, we found that the declined expression levels of *SOX-9, Col2a1*, and *Acan* in chondrocytes induced by IL-29 were dramatically alleviated by the treatment with Linagliptin, which was then pronouncedly abolished by the knockdown of Nrf2. These findings suggest that the protective effects of Linagliptin on the expressions of SOX-9, *Col2a1*, and *Acan* against IL-29 were dependent on Nrf2.

## Discussion

IL-29 is composed of a signal peptide (22 amino acids) and a mature peptide (178 amino acids), named interferon-λ (IFN-λ). IL-29 has been recently reported as an important pro-inflammatory factor involved in the pathogenesis of multiple diseases. Lin claimed that IL-29 induced inflammation and insulin resistance in obese animals [25]. In rheumatoid arthritis, IL-29 is also reported to mediate the lipopolysaccharide (LPS)-induced inflammation [26]. Recently, the involvement of IL-29 in OA development has been claimed [16]. ECM degradation, such as the declined expressions of collagen and aggrecans, is reported to be an important inducer for the development of OA symptoms [27]. We found that *Col2a1* and *Acan* genes were significantly downregulated by IL-29, indicating a potential effect of facilitating the degradation of cartilage ECM. These preliminary data are supportive of the establishment of an injury model on chondrocytes using IL-29 to simulate the symptoms of OA. As mentioned above, Linagliptin has been demonstrated to exert anti-inflammatory properties in various studies [13–15]. Therefore, we assumed that Linagliptin might have a protective effect on the development of OA.

Lefebvre [28] firstly reported a positive correlation between the expression levels of SOX-9 and *Col2a1* in the progression of cartilage formation, which are highly expressed in chondrocytes, indicating that *Col2a1* might be positively regulated by SOX-9. It has been recently reported that SOX-9 is significantly downregulated in cartilage tissues in OA, and plays an important role in maintaining the homeostasis of cartilage ECM [29]. In the present study, we found that the declined expression levels of *Col2a1* and *Acan* in chondrocytes induced by the stimulation with IL-29 are accompanied by the downregulation of SOX-9, which is consistent with the IL-1β-induced *in vitro* OA model described previously [30]. We further verified the significant function of SOX-9 in IL-29-treated chondrocytes by transfecting the lentiviral SOX-9, which dramatically upregulated the expression levels of ECM components. Our data confirm the involvement of SOX-9 in protecting the impairment of ECM against IL-29.

The expression level of DPP-4 was found to be significantly elevated by stimulation with IL-29, which reveals the potential protective effects of the DDP-4 inhibitors against IL-29-challenged chondrocytes. In the present study, Linagliptin, a novel DDP-4 inhibitor applied for the treatment of type II diabetes, was used to explore potential therapeutic effects against OA. We found that the syntheses of ECM components and expression of SOX-9 in IL-29-stimulated chondrocytes were significantly enhanced by the introduction of Linagliptin, indicating an obvious protective effect on damaged chondrocytes. Nrf2 is an important transcriptional factor involved in the regulation of the balance between the oxidative and anti-oxidative systems [31]. Dissociative Nrf2 is separated from the Keap1-Nrf2 complex to be transferred to the nucleus, activating the antioxidant reaction element (ARE) to induce the production of anti-oxidative or anti-inflammatory factors [32]. In the present study, we found that the level of nuclear Nrf2 was significantly declined in IL-29-treated chondrocytes, which was greatly reversed by Linagliptin, indicating its potential protective effect against the oxidative system. In addition, after knocking down the expression level of Nrf2 in chondrocytes, the protective effects of Linagliptin on the expressions of SOX-9, *Col2a1*, and *Acan* were dramatically abolished. These data indicate that Linagliptin might protect chondrocytes from inflammatory factors stimulation by activating the Nrf2 signaling pathway. However, the interaction between Nrf2 and SOX-9 needs further investigation in our future work. The other limitation is that we only used an *in vitro* cell model to examine the protective effects of Linagliptin on the reduction of ECM. In the future, more studies with both *in vitro* and *in vivo* models will provide more evidence to further clarify the effects of Linagliptin on OA. In addition, the direct target of Linagliptin in chondrocytes, as well as the underlying regulatory mechanism between Linagliptin and Nrf2 will be further explored.

## Conclusion

Our data show that IL-29 exerts a strong reduction effect on ECM by reducing the expressions of Col2a1 and Acan, which was reversed by Linagliptin. Mechanistically, we demonstrate that the protective effects of Linagliptin against IL-29-induced reduction of ECM in C-28/I2 chondrocytes were mediated by the Nrf2/SOX-9 axis. These findings suggest the potential application of Linagliptin in the treatment of OA.

## Data Availability

The data that support the findings of this study are available from the corresponding author upon reasonable request.
